# Emotional and Spiritual Challenges of Informal Caregivers: The Need for Early Mobile Palliative Care Support

**DOI:** 10.3390/nursrep15090327

**Published:** 2025-09-08

**Authors:** Samir Husic, Bojan Miletic, Tamara Stemberger Kolnik, Vedrana Vejzovic

**Affiliations:** 1 Department of Palliative Care, Health Centre of Primorje-Gorski Kotar County, 51000 Rijeka, Croatia; 2 Department of Clinical Medical Sciences, Faculty of Health Studies, University of Rijeka, 51000 Rijeka, Croatia; 3 Department of Postgraduate Studies, Faculty of Health Sciences, 3000 Celje, Slovenia; tamara.stemberger-kolnik@fzvce.si; 4 Department of Care Science, Faculty of Health and Society, Malmö University, 21109 Malmö, Sweden; vedrana.vejzovic@mau.se

**Keywords:** informal caregiver, palliative care, mobile palliative care team, oncology, qualitative, support

## Abstract

**Background/Objectives**: Informal caregivers play an important role in the palliative care of terminally ill family members at home. However, they often lack sufficient professional support, particularly in coping with emotional, spiritual, and practical challenges. This study aimed to explore caregivers’ experiences of providing care to a terminally ill family member at home. **Methods**: A qualitative approach was used to collect information from informal caregivers. Data were collected through interviews, and thematic analysis was used to identify the main challenges, coping mechanisms, and perceptions related to palliative care. **Results**: The study resulted in three themes: Involvement of professionals; The reality and dignity of death, and Life after death. Caregivers often associate palliative care exclusively with the last days of life, leading to delayed involvement of the mobile palliative care team. They stated that they preferred home care to hospital care and associated this with more positive experiences at the end of life. However, many reported feelings of loneliness and emotional distress, indicating a lack of appropriate psychological support. Spiritual care is often confused with religious practices, indicating a need for further education. **Conclusions**: The early involvement of mobile palliative care teams improves coping, facilitates a more humane dying process at home, and can reduce the emotional burden on caregivers.

## 1. Introduction

In the past, palliative care was generally only introduced when death was considered inevitable [1]. Today, it is widely recognized that palliative care should begin earlier in the course of a progressive illness. The word palliative is derived from the Latin word pallium, meaning mask or cloak. It refers to care that either “masks” the symptoms of a terminal illness or provides comfort when curative treatment is no longer effective [1].

One of the main aims of palliative care is to treat symptoms, particularly physical and psychosocial problems such as pain, breathlessness, fatigue, anxiety, and depression [2]. For patients with preserved autonomy, the focus is on alleviating physical symptoms, while patients with declining function require more emotional, spiritual, and social support [2]. Early palliative care should also take into account the burden and needs of caregivers [3].

Today, most patients with terminal illnesses wish to die at home surrounded by their family and with appropriate home palliative care, a preference supported by healthcare systems in many countries [4]. Both the European Association for Palliative Care (EAPC) and the World Health Organisation (WHO) advocate home-based, family-centered palliative care and emphasize the active role of the family in end-of-life care [5].

Informal caregivers (ICs)—often family members—are defined as people without formal healthcare training who care for someone with functional limitations, long-term psychiatric or physical illness, or age-related problems [6]. Knowledge of a poor prognosis and lack of cure can lead to acceptance of death, although many patients still pursue oncological treatments to delay confronting this reality [7].

The involvement of family caregivers in decisions to continue or discontinue treatment varies widely, from a passive to a dominant role and from a supportive to an obstructive one [8]. Often, ICs themselves need support during this process [8]. During active treatment, families usually follow the recommendations of oncologists. However, when oncologists recommend stopping an ineffective treatment, patients and relatives may resist and cling to any intervention that could prolong life [9]. At this point, when life-prolonging treatment is no longer an option, palliative care becomes essential for maintaining quality of life [10].

Despite its benefits, palliative care is often misunderstood as “the beginning of the end” [11]. ICs often feel unprepared for this stage and lack information about death, dying, and palliative services [12]. Research shows a strong correlation between the suffering of patients and their ICs—they act as an emotional system that influences one another [13]. With the recognition of the right to palliative care as a human right, the notion of a ‘good death’ or dying with dignity has become more important [14]. In this context, the IC, in collaboration with mobile palliative care teams (MPTs), plays a central role in facilitating a dignified death [15].

A partnership between ICs and palliative professionals is key to quality care and also to bereavement support and identifying pathological grief [16]. Whilst families are crucial to home care, ICs themselves can be seen as ‘second patients’ who need support [17]. Collaboration with MPTs is crucial to prepare ICs to provide effective care and to provide them with honest, timely information about disease progression and impending death, because “while the truth hurts, the deception can hurt even more” [18].

Spiritual support is also important as it helps ICs to find meaning, deal with uncertainty, and overcome feelings of hopelessness [19]. It provides emotional anchoring during critical life transitions such as illness and loss [20]. Many ICs report that spirituality and spiritual care provide patience, comfort, and a sense of security [21].

The family is recognised as an important partner in palliative care, as emphasised in the study by Tripodoro et al. [22]. However, many ICs report that they feel inadequately trained for certain tasks, such as symptom management, administering medication, or recognising signs of deterioration, as shown in several studies, such as a study by Li et al. and Chen et al. [23,24]. In addition, they often feel isolated and overwhelmed as the professional support they receive from the ICs’ perspective is often inadequate, and guidance is unclear. ICs are expected to make important decisions and perform complex medical and non-medical tasks [25]. Without adequate professional guidance, these tasks can lead to uncertainty and fear of making mistakes. As a result, ICs’ stress levels increase, leading to emotional exhaustion and a possible feeling of helplessness [26]. A long period of caring can also lead to burnout [27]. Ultimately, informal caregivers can develop long-term consequences for their own physical and mental health, such as anxiety, depression, and chronic fatigue [28]. All of these factors have a negative impact on the quality of palliative care. As patients and ICs form an emotionally interdependent system, their needs and concerns should be addressed together. Understanding caregivers’ experiences is therefore essential to ensure comprehensive, early, and dignified palliative care based on strong partnerships between families and professionals.

Although spirituality is central to holistic care, the spiritual needs of family caregivers remain under-researched. Most of the existing literature focuses on patients, while leaving aside caregivers’ spiritual struggles, their sources of resilience, and their experiences of finding meaning. Qualitative studies in this area are particularly lacking, although they have the potential to deepen understanding of this important but often neglected dimension of caregiving.

The aim of this study was therefore to explore caregivers’ experiences of providing care to a terminally ill family member at home.

## 2. Materials and Methods

This article is part of a wider research project exploring the experiences of ICs providing palliative care to family members at home. A qualitative, exploratory research design was used in which in-depth semi-structured interviews were conducted in the homes of ICs. The study focused on obtaining rich verbal accounts that provided a comprehensive understanding of participants’ experiences [29], particularly the complex and often overlooked phenomena involved in caregiving for people in the palliative phase. Data were analyzed using reflexive thematic analysis [30], and the research protocol was developed following the Consolidated Criteria for Reporting Qualitative Research guidelines [31]. To strengthen the credibility of the findings, caregiver quotations are used in the Section 3 to illustrate how themes were grounded in the data. The analytic process was also discussed with academic peers familiar with palliative care research to ensure coherence and confirmability. In addition, member checking was conducted, as two participants reviewed the data and confirmed the researchers’ interpretations throughout the analytic process.

### 2.1. Study Setting

The study was conducted in Primorje-Gorski Kotar County, Croatia, a region where people with advanced illness have the opportunity to receive home-based palliative care provided by a multidisciplinary team. This team provides comprehensive care, including pain management, treatment of concomitant symptoms, and psychosocial support for patients and their families. Within the Croatian palliative care context, the role of the MPT regarding spirituality was to provide supportive presence and to respect the existing religious or non-religious beliefs of patients and caregivers. The team did not apply structured spiritual interventions, nor did it seek to influence or convert participants’ faith. Instead, spiritual care was expressed through empathetic communication, attentive listening, and openness to caregivers’ own sources of meaning.

### 2.2. Participants

A total of 12 adult ICs (aged 18 years or older) were included in the study. Each of them had cared for a family member who was receiving palliative care at home until their death. Recruitment took place between December 2023 and May 2024 and was supported by regional healthcare professionals. Inclusion in the study was only permitted after the Ethics Committee of the Primorje-Gorski Kotar County Health Centre had given its approval (ref. no. 01-286/1-2-23, issued on 9 May 2023). All invited participants gave written informed consent.

For this study, an informal caregiver was defined as an unpaid person, usually a family member or friend, who provides physical, emotional, and practical support to a person with a life-limiting illness. This support may include helping with daily tasks, monitoring symptoms, administering medication, coordinating care, and providing companionship.

### 2.3. Data Collection

The interviews were conducted by the first interviewer, who has professional experience in palliative care. The last author, an experienced qualitative researcher with experience in nursing, methodically supervised the process. A semi-structured interview guide was used to provide consistency across interviews while allowing participants the flexibility to share their personal experiences in depth. The guide included open-ended questions focusing on caregivers’ experiences of providing care, their perceptions of professional and spiritual support, and the impact of the multidisciplinary team. The first interview served as a pilot to refine the questioning technique and was excluded from the data analysis.

At least one month after the patient’s death, a nurse from the MPT contacted the ICs, informed them about the study, and gave them time for reflection and questions. Once written consent was obtained, the interview was scheduled according to the participants’ time and location preferences. All interviews were recorded using an offline device. Interviews began with a collection of demographic data and were guided by a prepared interview guide. Additional open-ended questions such as “Could you elaborate on that?” and “Can you tell me more about that?” were used to elicit more in-depth responses. The duration of the interviews ranged from 16 to 46 min, with an average length of 25 min.

### 2.4. Data Analysis

We applied reflexive thematic analysis, as developed by Braun and Clarke [32], to explore informal caregivers’ perceptions and experiences. This method was chosen because it enables a flexible yet systematic approach to qualitative data analysis, while acknowledging the active role of the researcher in the co-construction of meaning rather than assuming a purely passive or objective stance. Reflexive thematic analysis was particularly suited to our study as we aimed not only to identify recurring patterns in caregivers’ narratives but also to explore the underlying meanings and contextual nuances related to their perception of MPTs. The first author transcribed the interviews verbatim. Both the first and last authors listened to the full recordings to develop an initial understanding of the material, and both authors independently reviewed the transcripts several times to familiarize themselves with the data through repeated reading of the transcript and created preliminary codes inductively and iteratively. These codes were then compared and discussed between the researchers who independently prepared the codes. Further analysis was conducted with the entire author team, which comprised professionals from diverse disciplinary backgrounds, including physicians and nurses. This approach ensured a multi-perspective interpretation and helped to minimize potential researcher bias. Through iterative discussions based on the identified codes across the entire dataset, potential subthemes and overarching themes were defined and named. The final report was developed on the basis of consensus reached among the participating researchers. An analytical framework illustrating Subtheme 1. A—Support of Multidisciplinary Palliative Teams (MPTs) at Home is present in Table 1. It presents the process of qualitative analysis, demonstrating how individual quotations were coded and grouped into subthemes that reflect the experiences, challenges, and evolving perceptions of patients and informal caregivers regarding the involvement of MPTs in home-based palliative care.

## 3. Results

### 3.1. Theme 1–Involvement of Professionals

Insufficient knowledge of both the disease and the patient’s care affects the confidence of ICs, who are unsure whether the care they are providing is beneficial to the patient. The support of trained healthcare professionals can be a prerequisite for the patient’s well-being, especially when the patient knows the final outcome of the disease.

MPTs must ensure regular monitoring of the patient and consistent support for the caregiver until the end of the patient’s life.

The negative perception of palliative care by both the patient and the IC can be completely changed by such an approach by healthcare professionals. According to caregivers, this comprehensive support reduced fear and uncertainty and allowed them to perceive their family member’s final "days as more dignified and peaceful. In this way, the role of the MPT was described by caregivers as contributing to what they understood as a “good death”. Caregivers described different trajectories in their spiritual experiences during and after the death of their family member. For some, existing faith was a source of strength and meaning, helping them to endure the emotional burden of caregiving. Others, however, reported feelings of doubt, unanswered questions, or even spiritual distress, reflecting the complexity of existential struggles in the context of end-of-life care. A few caregivers stated that their spiritual outlook remained unchanged, emphasizing instead the importance of professional and emotional support. These diverse experiences illustrate that spirituality can be both a resource and a challenge in the caregiving process.

#### 3.1.1. Subtheme—Support of MPTs at Home

Patients and ICs often associate palliative care with the end of life, which is why they either reject the MPTs or are reluctant to call them.

The partnership between the caregiver and the patient ensures better collaboration between the dyad (patient/caregiver) and the healthcare team and helps to change participants’ negative perceptions of MPT visits.

*Wife (IC 09): “When I told him (my husband) ‘palliative’ he said, ‘But I’m not dying’ /…/ and I said it’s not about dying, they’re great…. B. spoke highly of them…. let’s call them… come on… and that’s how it started.”*

Some caregivers in our study found out about MPT via the internet or from acquaintances who were better informed or already had experience with MPT.

*Wife (IC 09): “A friend (B.), who was unfortunately in a similar situation to us, told us about the palliative care teams /…/ they had the same palliative care team visiting them and he recommended them to us.”*

The most common reasons for MPT visits are physical symptoms (pain, vomiting, dyspnea). Conversations during visits open the door to addressing other issues and help to build the confidence of both the patient and their family.

*Wife (IC 09): “He was in pain, vomiting frequently, and had a terrible cough. So I rang you. And that’s how you started working with him. I remember him saying: ‘That’s it, you’re going to treat me until I die’ /…/ and that’s how it was.”*

The organization of out-of-hospital palliative care meant that nurses were sometimes forced to call the emergency services in crises.

*Husband (IC 05): “The emergency team came once or twice when we had to call— at weekends when you weren’t available. We didn’t call them often; we had you whenever we needed you.”*

Caregivers who have used MPT’s services for some time prefer to communicate with them rather than the emergency services or other agencies. MPT’s ongoing support provides a better understanding of both the patient and the caregiver, which leads to more effective problem-solving. In our context, the role of the multidisciplinary team (MPT) regarding spirituality was not to provide a structured religious intervention or to promote any particular faith. Instead, caregivers reported that the team offered emotional presence, attentive listening, and openness to their personal beliefs and doubts. This approach helped caregivers to reflect on existential issues and to find comfort in accordance with their own values. Importantly, no attempt at religious conversion was undertaken, as the focus of the MPT was to respect and support the caregivers’ individual spiritual perspectives.

#### 3.1.2. Subtheme—Spiritual Support

The approaching end of life increases the need for support in relation to the emotional and spiritual struggles of both the patient and the caregiver.

Reducing spiritual care to religious practices can lead to the need for spiritual support being dismissed or misunderstood.

*Daughter (IC 12): “Z. (the patient) was not a believer, and neither are we… so we didn’t feel that we needed such support, and that’s why we didn’t ask for it.”*

In some cases, a dominant member of the patient–caregiver dyad imposes their views and neglects the actual needs of the patient.

*Patient’s husband (IC 02): “It’s not in my nature, so there was no need for spiritual support.”*

In emotionally difficult moments, family members may express disbelief and anger or question God’s will and mercy.

*IC 02: “I don’t know what to tell you /…/ people usually say, ‘God, what did I do to deserve this?’ /…/ Yeah, there’s not much mercy from Him.”*

Sometimes, a lack of understanding of spirituality leads to ‘unusual’ actions by caregivers who look to other religions or forms of prayer for a lifeline. Whether this makes sense or not was rarely discussed in the dyads.

*Husband (P 05): “We are not religious. Well, we celebrate Christmas and Easter more because of the family connection. We have prayed for them in churches and mosques. We even got Viputi from Sai Baba. We go to everything. Whatever helps.”*

As death approaches, attitudes towards spirituality can change. The presence of spiritual support can become a significant source of comfort and relief.

*Wife (P 06): “He (the patient) wanted to go to Marija Bistrica. We travelled there for two days, prayed in the church, just the two of us, and cried. When medicine can’t help, you have to believe in something.”*

In prayer, both the caregiver and the patient often found peace, relief, spiritual renewal, a feeling of liberation, and God’s protection.

*Wife (IC 09): “Then we put everything in God’s hands. We went to a church in B. where he… I don’t know what… he cried so much, and so did I. He said: This is a special kind of peace, a spiritual renewal.”*

Understanding the spiritual needs of spouses or recognizing significant family moments (such as the birth of a young child) appears to be an important factor in prolonging and improving the quality of life.

The “statistical estimates” of doctors on life expectancy often lose their significance.

*Wife (IC 09): “They (the doctors) gave him three months to live, but he lived another year and a half (smiles). He realized that only God decides when a person’s time is up. Our little one (son) gave him a lot of strength to live longer.”*

For patients who are believers, faith in God and the support of clergy can make it easier to deal with end-of-life difficulties.

*Daughter (IC 08): “I’m sure that the spiritual support meant more to her than the medical help. For her, it was also psychological support. Yes, I think it was.”*

Shared prayers involving the priest and the whole family (patient, caregiver, and children) are an example of the spiritual care that should be made available to those in need.

*Wife (IC 09): “A priest came to him, he confessed and felt better…. That day, he (the patient) didn’t wake up. The children and I held hands in a circle. He (the patient) opened his eyes. The priest said: Now I have learnt what spirituality means. He looked at me… he is dying.”*

Meditation, spiritual counselling, and other forms of engagement with the spiritual dimension—both for the patient and the caregiver—can be very helpful in the dying phase.

*Husband (IC 10): “S. was not a traditional believer, but she had a spiritual counsellor who visited us. We had meditative conversations and stayed in touch with that spiritual dimension, and that was helpful.”*

As the end of life approaches, caregivers often begin to question the meaning of life and death. The loss of a shared life and plans for the future can cause them to question God’s choices.

Some become sad or angry with God and reject spiritual support, while others—either of their own accord or at the suggestion of the caregiver—accept prayer and usually feel relief and peace.

Those who were already religious tend to pray more and benefit from visits to priests and prayers together.

### 3.2. Theme 2—The Reality and Dignity of Death

The time immediately before and after death is one of the most difficult and emotionally sensitive moments for caregivers.

As the patient’s end of life approaches, a new challenge arises for healthcare professionals in mobile palliative care teams: they must prepare the caregiver to recognize and accept death as inevitable.

Caregivers are often reluctant to actively think about death and the dying of their loved ones.

The descriptions of the last day of our participants’ lives were full of intense emotion and the conviction that the last interaction with their loved one was a final goodbye before death.

#### 3.2.1. Subtheme—When the End of Life and Death Become Real

Analysis shows that caregivers’ reactions differ depending on whether the patient died at home, with the caregiver present, or in a facility (hospital, hospice). It was important to caregivers to fulfill the patient’s wish to die at home. Some caregivers expressed a “wish or prayer to God” for the difficult final days to end quickly. In prayer, they sought comfort.

*Husband (IC 03): “She was fading minute by minute, and that was the end. Considering everything we had been through, honestly, I could only pray to God to take her.”*

Descriptions of the final moments of life—or the time immediately after the patient’s death-were sometimes filled with elements that appeared “unreal,” or were the caregivers’ perceptions.

*Husband (IC 05): “In the last 4–5–6 days, she was fading, then would return for five seconds. It was obvious she was slipping away. Then the younger son came, and she kissed us. We just said, ‘Mom is here, Mom knows.’”*

*Wife (IC 09): “And he died with a smile. I looked at him and said, ‘Thank you, God,’ and he smiled.”*

Caregivers often hoped that the patient still had more time left (“there is always time for death”), while also feeling conflicted about whether death came too quickly, and whether that was good or bad for the patient and themselves.

*Daughter (IC 08): “It all happened so quickly. I know she went without pain, that she just fell asleep. Is that good or not? I think it’s easier for the patient to go that way. And for the family, too.”*

Caregivers who had the support of MPT felt more secure and better prepared to understand the dying process, which helped them find peace in continuing life afterward.

*Wife (IC 09): “I remember you (MPT) telling me: now it’s your turn. And I thought—what do you mean, my turn? You said: We’re preparing you for what’s coming. But you can’t ever really be prepared for that moment. He was lying there, dying. He was calm, breathing peacefully, and just went. That gave me the peace I live with now.”*

Death in an institution sometimes leaves caregivers with unresolved questions and uncertainties about the dying process—occasionally even a disturbing feeling that the death happened “somewhere out there,” in a “bad way,” where the patient may have been afraid or alone.

Still, some caregivers felt safer when the patient was in a healthcare facility.

*Husband (IC 10): “There (in the hospice), she felt alone. She was alone in the room. Yes, people visit, but you’re alone. She was overwhelmed with fear, terrified.”*

The unfulfilled wish to bring the patient home “to die in the peace of their own home” sometimes became an additional emotional burden for the caregiver after the patient’s death. Some caregivers lacked trust in healthcare professionals working in institutions, expecting a different approach and better quality of care.

#### 3.2.2. Subtheme—The Dignity of Death from the Caregiver’s Perspective

Dignity of death is most often described by caregivers as a “peaceful death” or “the patient fading away” without restlessness, pain, or other distress that often accompanies patients near death. Care provided by the family brings the patient a sense of peace, security, and love. Fulfilling the wish to stay and die at home is one of the aspects of a dignified death from the patient’s perspective. Patients who spend their final days outside their home have difficulty adapting to the unfamiliar environment. They experience feelings of insecurity and fear, which leave caregivers with lasting emotional scars and a sense that they have not done everything they could. They ask themselves: How did they die? Were they alone? Did they suffer? The wife describes her husband’s death, considering it dignified:

*(IC01) “I think he died with dignity. I was beside him. I saw that last breath; it was like a puff of steam coming out of his mouth, like white air. And then I saw he wasn’t breathing. He just faded away.”*

Both patients and caregivers often expressed the desire to be together until the end. The husband *(IC03)* recalls a conversation with his wife about staying in their home until the end of her life:

*“I promised her I would be with her until the end; that was also her wish. I came home and said: ‘Don’t worry, K., you’ll be home until the end.’”*

Family support at these moments eased the patient’s death and brought satisfaction to the caregiver who fulfilled that wish. The patient’s daughter *(IC12)* shares a similar sentiment:

*“It was much better that he died at home, although it’s hard for the family. We wanted him to be here, to see us. If he had died somewhere else, God forbid. ”*

A similar experience is shared by a wife *(IC09)* who believes it is very important to ensure the dignity of death, conveying this as a message to other patients and families:

*“Yes, it was a dignified death. I’m so glad he stayed at home; he was with me. And I tell everyone: this is my experience, my peace. I now live with the fact that I did everything in my power.”*

According to caregivers, dignity in death is not only the absence of pain and physical suffering but also the absence of fear that accompanies the end of life. The husband of a young patient *(IC10)* says:

*“Hm, I don’t even know how to understand dignity? She died in great fear. She had no pain, but it’s not just pain, you know, it’s what makes life hard. I don’t know what was missing; it seems to me that someone to deal with the fear was missing. They all take care of the body, you know.”*

Some caregivers leave the patient in an institution, expecting that the medical staff will “ensure” all aspects of dignity at the end of life, but express anger or dissatisfaction with healthcare professionals. A dominant husband states: *(IP02)*

*“The dignity of such a death was very questionable. Then that night, I was called to say that my wife had passed away. You get into a situation where you bring such a patient to an institution that should, in such situations, ease the passing of anyone, so they die with dignity. I’m not sure.”*

### 3.3. Theme 3—Life After Death

Caregivers may be prepared for the emotional aspects of loss but not the practical, or vice versa; rarely are they fully prepared. Some dared to talk about the ‘events’ after death while the patient was still alive, discussing the funeral arrangements or the procedures that followed, but most could not, referring to the many years they had spent together. The room in which the patient died was often a ‘room of memories’ where caregivers did not want to change anything for a while, perhaps out of a desire to keep the memory ‘in that room for as long as possible’ “ The stress caused by the long and intensive care, psychological pressure and grief can suddenly subside after the patient’s death and manifest itself in different ways.

#### 3.3.1. Subtheme—I Must Continue Living After the Loss

The analysis shows that the caregiver was ready for the pragmatic component of the loss of her husband (e.g., talking about the funeral) but still showed emotional unreadiness to fully accept his death. The wife recalls: *(IC 01)—“When he was going for surgery, we needed to talk to him (the patient)… what if something bad happens. I didn’t have the courage for that conversation. We had been together for 45 years”.*

As time passes, the caregiver often remains alone, left with their memories, insomnia, thoughts, and “sounds coming from the patient’s room.” The wife *(IC 01)* said: *“In the first days after his death, there were many obligations, including the funeral. Later, when I was alone, I knew I wouldn’t sleep all night. I would hear someone calling me, go to the room, and recite the Fatiha (prayer) for the soul of the deceased”.*

Caregivers kept and cared for the belongings that belonged to the patient. The husband describes his emotions after the death of the patient: *(IC 03) “The door to her room was closed for a long time, certainly for 2 months we didn’t touch anything. The cup, her lip balm, were untouched”.*

Physical ailments combined with emotional ones, loneliness, and avoidance of people were very pronounced in some caregivers. The wife explains: *(IC 06)–“The funeral passed as if I wasn’t there. Then, when my body started to relax, I had pain all over, all my muscles ached. When I locked myself at home, that was the hardest for me”.*

Distraction, moments when one forgets for a while what “cannot be forgotten,” time that still “does not heal everything”—these are all factors that help the caregiver continue their life. The wife *(IC 06)* said: *“And then I said, I’m going to work because I would go crazy at home. It’s been 3 months now. It’s hard, I’m alone, I miss him. Our son lives here, but he has his own life. It’s good for me to have responsibilities, to have to cook. Responsibilities are good therapy”.*

Friends and children proved to be very helpful in overcoming the constant thinking about the deceased husband, about death in general. The wife *(IC 09)* said: *“People, those people around me, bothered me. Today, I’m grateful to them. Friends call and ask: what did you cook today, what shall we do now? That’s how the days passed”.*

The emotions after the wife’s death made the caregiver tired, longing for rest and sleep, opposite to the desire for life. He considered it a terrible “mess.” The husband: *(IC 10)–“(Sighs–pause) … when she died, I was exhausted, emotionally destabilized, sadness, despair, something that constantly wells up. On the other hand, I had some desire for life, to continue as before. It’s chaos, a total mess”.*

Support from friends, family, returning to work, and family obligations, for most caregivers, has a good “therapeutic effect” in their struggle to return to “normal” life, integration into their surroundings, and resocialization.

#### 3.3.2. Subtheme—Would You Do It the Same Again? (Related to the Patient’s End of Life)

Analyses show that caregivers reflect on “whether “they have made mistakes, whether they could have been more careful. When they receive “confirmation” from the MPT that they have done everything well, they feel relieved and unburdened. Institutionalization was an unacceptable option for most caregivers who had a partnership relationship with patients.

The wife says: (IC 01) “He wanted to stay at home. I asked him if he wanted me to put him in a hospice, and then he started refusing to eat and drink. I wasn’t in favor of that either. I tried to give him the best care I knew how; I think we owe it to each other. Now I’m calmer; my conscience doesn’t bother me anymore”.

The wife *(IC 07)* emphasizes the importance of the support she received from healthcare professionals, which she sees as a key reason for the decision to care for the patient at home: *“No, we wouldn’t have put him in a hospice. I would still have left him at home and cared for him. I think that was good and the best thing for him. I tried, with your (MPT’s) help; I couldn’t have done it on my own.”*

The wife *(IC 09)* shares her thoughts. She believes that everything in life happens “for a reason,” according to God’s will. “If it were up to me, I would sign such a death, with my children with me, with all my loved ones. And that I am at peace. In my own home and with palliative care. You (MPT) were sent for a reason; it’s all arranged by God”.

Caring spouses usually remember with wistfulness the beautiful moments of their life together and the support they gave each other in difficult times. The husband *(IC 03)* says: *“You see, K. and I never argued, despite all the problems we had. Out of 365 days a year, we spent 300 days in the hospital (her other daughter died of a tumor in the hospital). We had no time for nonsense. Our lives were turned upside down”*.

The dominance of either the caregiver or the patient led to the caregiver’s dissatisfaction after the patient died. The husband *(IC 02)* of a patient who died in the hospital believes that death in such an environment is bad for the patient. For him, this later caused stress and regret: *“I would have taken my wife home to finish at home, 100%. At least she would have been at home; we said goodbye to all the deceased from our families from home.”*

A caregiver who was not prepared to take on the full burden of home care and who did not cope well in this care process has a different opinion. The husband *(IC 10)* says: *“Would I have done anything differently? No, I wouldn’t have taken her out of the hospice. I think it was better for her to be there. It was easier for all of us. I mean, she was making decisions all the time; I couldn’t force her to do anything.”*

Most caregivers whose patients have died at home say that with the support of the palliative care team, they would repeat the “same scenario” of caring at home. They say that the intensity of grief is less in such circumstances. They emphasized that the MPT did not impose any religious views but created space for caregivers to express their own beliefs and doubts. This respectful approach helped caregivers find meaning and comfort in accordance with their personal values, which they perceived as an important form of spiritual support. On the other hand, if the patient died in an institution, they show a greater intensity of anger, aggression, and guilt.

## 4. Discussion

The findings of this study aimed to explore caregivers’ experiences in providing care to a terminally ill family member at home.

Our results suggest that early involvement of the MPT—not just in the final days of life, as is often standard clinical practice—could enhance caregivers’ and patients’ preparedness for the dying process. However, a lack of understanding of the goals of such support—by both the caregiver and the patient—was identified as a barrier to its utilization. The results of the Breen et al. study suggest that even if caregivers are cognitively prepared, they are never fully prepared emotionally for this challenge. Palliative care tends to focus on preparing patients for death rather than preparing caregivers for the new situation or life after death [33].

Our study also showed that both caregivers and patients themselves often associate palliative care exclusively with the end of life. As a result, they may hesitate to refer to MPTs, or they may procrastinate. In times of crisis—especially when physical symptoms such as pain become severe—caregivers often turn to MPTs on the recommendation of friends or acquaintances who already have experience, rather than on the advice of healthcare professionals. This often happened too late in the course of the disease and outside of a formal referral process. This pattern may indicate a continued lack of awareness and education about the role and timing of palliative care, although there is evidence that early integration of palliative care—even alongside active antineoplastic treatment—improves the coordination of care for patients with advanced disease, as shown in the study by Ferrell et al. [34].

Research such as that by Johnson et al. also shows that oncologists refer patients to palliative care primarily because of physical symptoms and not in response to psychological or social problems [35].

In the Croatian setting at the time of our study, organizational limitations meant that in crisis situations, caregivers were often forced to contact emergency medical services. Caregivers expressed a preference for interacting with MPTs over emergency personnel, perceiving them as more supportive and aligned with the patient and family’s needs. On the other hand, early palliative care has been associated with less aggressive treatment in the final weeks of life and decreased use of emergency services and intensive care units [36].

We have found that a strong partnership between caregivers and patients can facilitate access to palliative care, promote better collaboration with healthcare professionals, and help to change negative perceptions about the involvement of palliative care in patient care. A continuum of coordinated care can help address the complex challenges faced by patients and their caregivers together. Our findings suggest that confronting the end of life and an uncertain future increases the need for emotional and spiritual support- both for patients and their caregivers. This is consistent with previous research, such as that of Gardner et al., which emphasizes that spiritual care provides support and recognition of people’s needs during major life transitions, including illness and loss [20].

Addressing spiritual needs in collaboration with trained professionals and following the wishes of patients and caregivers contributed to a sense of spiritual fulfilment in our study, regardless of previous attitudes towards spirituality. Benites et al. conducted a meta-synthesis on spirituality in caregivers of cancer patients receiving palliative care and found that caregivers expressed their spirituality in multidimensional ways. They concluded that exploring caregivers’ spiritual needs could enrich multi-professional psychosocial support [37].

In our study, caregivers often associated spiritual care exclusively with religious practices, which can lead to misunderstanding or rejection of the need for such support. However, the literature emphasizes that spiritual care should not be limited to religion—it should also include the promotion of hope, compassion, and responding to existential needs, helping individuals to find meaning in their experience [38].

The approach of death has changed the attitude of some of our caregivers towards spirituality. Sometimes they sought help from “other faiths and prayers”. They were aware that “they don’t have much time left”. Studies on this topic have reported that caregivers need spiritual care to feel hope, to be prepared for the death of their family member, and that caregivers find strength, comfort, and encouragement for continued care through spirituality, making it easier for themselves [39,40]. The concept of a ‘good death’ in this study should not be understood as an attempt to institutionalize or standardize dying. Rather, it reflects how caregivers themselves described their experiences when supported by a multidisciplinary team. Their accounts pointed to aspects such as relief from suffering, dignity, and emotional support, which they perceived as contributing to a meaningful and peaceful end-of-life process. In this sense, the notion of a ‘good death’ emerged from caregivers’ perspectives, not as a universal framework but as an individualized interpretation shaped by their lived experience.

The moments before and at the moment of death were an additional burden for the caregivers in this study. As the patient approached the end of life, caregivers often questioned the meaning of life and death. Some showed anger by rejecting spiritual support, while others who had not previously had a positive attitude to religion accepted prayers and felt relief and peace. Those who had previously been religious were more likely to use prayer. Caregivers often expect healthcare professionals to answer the question: How long will it last? When will it end? In our study, they often wondered whether death came too quickly, whether it was good or bad for the patient. Despite previous knowledge of caregivers’ needs, they still struggle with similar issues because contact with MPTs occurs late [41,42].

The results of our study show that most caregivers experience a deep sense of loneliness after the loss of a terminally ill relative, often accompanied by emotional difficulties and social withdrawal. These reactions are related to the intense stress that has accumulated during the caring period. This is in line with the findings of Madsen et al., who reported that caregivers have to redefine their role in daily life after a loss and that loneliness is a prevalent feeling during deep grief [43]. The emotional fluctuations that our participants described as a kind of “chaos” are consistent with Hooghe et al.’s dual process model of grief, which emphasizes the dynamic interplay between loss-focused and recovery-focused coping [44].

For some participants, the room in which the patient had died had a strong emotional meaning—it often became a ‘room of memories’. Boerner et al. observed that bereaved people often seek the presence of the deceased and maintain their daily routines, which can hinder effective loss-focused coping [45]. In contrast, other participants found that returning to work and daily responsibilities had a therapeutic effect by providing distraction and helping them to move on. This is consistent with Holtslander et al., who emphasize the importance of social support as a source of comfort and strength during grief [46].

Most of the caregivers in our study, whose relatives died at home, stated that they would choose the same care setting again with the support of MPT. In contrast, caregivers of patients who had died in a care home reported more intense grief. An Israeli study also found that over 90% of caregivers who had been cared for at home viewed the patient’s death as a positive experience for themselves and the patient, significantly more than those whose loved ones had died in a hospital. Over 95% stated that they would choose home care again in similar circumstances [47].

In conversations with MPT members, caregivers often reflected on their lives with the deceased and recalled shared moments and mutual support with a sense of nostalgia. O’Sullivan et al. found that 52.8% of caregivers felt adequately supported at the time of death, although 21% did not engage in follow-up conversations, despite the recognized value for grief processing [48].

Finally, one participant found that death in the hospital increased the patient’s anxiety and subsequently led to stress, sadness, and regret in the caregivers themselves. This experience aligns with the findings of Wang et al., who identified emotional support as the most common unmet need of patients, reporting that over 30% of ICs struggled the most with managing both their own and the patient’s emotions [49].

A key strength of this study lies in its in-depth exploration of the spiritual dimension of caregiving in palliative care. By focusing on how informal caregivers experience professional and spiritual support, the study sheds light on an underexplored but crucial aspect of end-of-life care. The use of reflexive thematic analysis further enabled a nuanced understanding of caregivers’ voices and perspectives, supported by rich qualitative data. These insights contribute original knowledge to the international literature, particularly by highlighting the intersection of professional support, cultural context, and spiritual needs.

At the same time, certain limitations must be acknowledged. The study was conducted in a specific cultural and healthcare context in Croatia, which may influence how spirituality and caregiving are understood and practiced. Additionally, the sample size was relatively small, which restricts the transferability of the findings. Nevertheless, the detailed accounts provided by participants offer valuable depth, and the study’s findings may serve as a basis for further research in different cultural settings and with larger samples.

In addition, cultural factors and differences in healthcare infrastructure may influence how palliative care is understood and utilized, further limiting the transferability of the results to other areas. Nevertheless, the insights gained from this study offer valuable perspectives that can be useful in developing and improving multidisciplinary palliative care teams (MPTs) both in this region and in similar healthcare contexts.

## 5. Conclusions

This study highlights the need for early identification and support of informal caregivers, who often face emotional exhaustion, spiritual distress, and a lack of preparedness when caring for patients with progressive illnesses. Many remain unaware of mobile palliative care services or associate them only with end-of-life care, which delays timely and beneficial support. Nurses and other health workers play a key role in recognizing caregiver burden and in providing early, non-judgmental guidance, particularly in addressing emotional and spiritual needs. Strengthening the role of nursing within MPTs and promoting interprofessional collaboration is essential to improving caregiver well-being and patient outcomes. Supporting caregivers must become an integral part of nursing practice, not only at the end of life but from the time of diagnosis onwards. This calls for a more structured, person-centered approach in nursing education, policy, and clinical care. Finally, this study underlines the importance of supporting the spiritual dimension of informal caregiving in palliative care. We recommend targeted training for healthcare professionals, policy-level efforts to integrate spiritual care into palliative care guidelines, and strengthening the capacity of mobile multidisciplinary teams. These steps could provide more holistic support for informal caregivers.

## Figures and Tables

**Table 1:** Example of the coding process: from initial codes to main themes.

Quotation (Participant)	Initial Code	Subtheme	Main Theme
“When I told him “palliative” he said “but I am not dying”	Negative perception of palliative care	Support of MPTs at home	How professionals can help us
“A friend told us about the palliative care teams”	Informal knowledge transfer about MPT		
“He was in pain, vomiting frequently…I rang you”	Physical symptoms trigger contact with MPT		
“The emergency team came,…when you (MPT) weren’t available…”	Limitations in the accessibility of MPT		

## Data Availability

The data are available on request from the corresponding author.

## Public Involvement Statement

Consolidated criteria for reporting qualitative research (COREQ) were used.

## Guidelines and Standards Statement

This manuscript was drafted against the COREQ (Consolidated criteria) for reporting qualitative research.

## Abbreviations

The following abbreviations are used in this manuscript:

IC	Informal caregiver
MPT	Mobile Palliative Care Team
